# Age-specific definition of low anti-Mullerian hormone and associated pregnancy outcome in women undergoing IVF treatment

**DOI:** 10.1186/s12884-021-03649-0

**Published:** 2021-03-05

**Authors:** Depeng Zhao, Jing Fan, Ping Wang, Xuan Jiang, Jilong Yao, Xuemei Li

**Affiliations:** Department of Reproductive Medicine, Shenzhen Maternity and Child Healthcare Hospital, Southern Medical University, Shenzhen, China

**Keywords:** Anti-Mullerian hormone, Age, In vitro fertilization, Pregnancy

## Abstract

**Background:**

The age-specific definition of low anti-müllerian hormone (AMH) is lacking. This study aims to define an age-specific reference for low AMH and to evaluate the associated outcome in women undergoing IVF treatment.

**Methods:**

A retrospective study was performed in women receiving IVF treatment at the Shenzhen maternity and child healthcare hospital between September 2016 and September 2018. We excluded cases without AMH concentration. Polynomial least-squares regression was used to estimate the age-specific reference ranges for AMH after log-transformed. The age-specific 10^th^ centile was defined as the threshold of low AMH concentration.

**Results:**

A total of 909 patients were analyzed in this study. The age-specific reference ranges for AMH were established using linear regression model and resulted in an age-specific equation for mean: mean of LnAMH = (− 0.085 × age) + 3.722 (ng/ml, in unit). Women with AMH level higher than 10^th^ centile had favorable outcomes in ovarian stimulation compared to those with low AMH level. In patients younger than 35 years, the rates of clinical pregnancy per transfer and ongoing pregnancy per transfer in the subgroup with AMH level higher than 10^th^ centile were significantly higher than that in the subgroup of low AMH level, 57% versus 31.3% *p* = 0.003 and 51.9% versus 21.9% *p* = 0.001, respectively.

**Conclusion:**

Women with AMH lower than age-specific 10^th^ centile had significantly unfavorable outcomes after IVF treatment. The age-specific 10^th^ centile of AMH concentration may be useful to predict the outcome of IVF treatment.

## Background

The anti-Mullerian hormone (AMH) belongs to the superfamily of transforming growth factor-β (TGF-β) and is mainly expressed in granulosa cells of small follicles ≤8 mm diameter [1]. In ovarian folliculognensis, AMH inhibits the primordial to primary follicle transition and limits the number of early atrial follicles which will develop into dominant follicle for ovulation [2]. The serum AMH level thus well corresponds to the number of atrial follicles in ovaries [3]. As a result, AMH is widely applied to test the ovarian reserve in women undergoing IVF treatment and recently to predict pregnancy outcome after assisted conception [4]. Several retrospective studies reported a positive association of AMH level with pregnancy rates after IVF treatment [5, 6]. These findings were confirmed in a large prospective study and a metaanalysis [7, 8]. Nevertheless, other studies reported a weak or not significant association between AMH level and pregnancy outcome [9–11]. Therefore, AMH may be a potential predictor for pregnancy outcome after assisted conceptions, but further investigation is still warranted.

The serum AMH level decreases steadily with advancing age. Most previous studies however used an arbitrary cut-off value from 0.4 to 2.7 ng/ml based on the method used for assay to differentiate pregnancy outcome [12]. The AMH level varies greatly in women at same age [13, 14]. A universal threshold of AMH level may result in that the majority of women at advanced age are classified into the low AMH group, subsequently leading to a poor pregnancy outcome after IVF conception. In addition, maternal age after 35 years is strongly related to embryonic aneuploidy, increased miscarriage rate and decreased live birth rate [15]. The age-specific cut-off value of low AMH could be thus useful to investigate the association of AMH with pregnancy outcome in assisted conceptions. The purpose of this study is to establish an age-specific cut-off value for low AMH level and to investigate the association between low AMH level and pregnancy outcome in a large cohort of women undergoing IVF treatment.

## Methods

### Study subjects

All women undergoing IVF treatment at the Department of Reproductive Medicine, Shenzhen Maternity and Child Healthcare Hospital from January 2016 to October 2018 were included in the present study. Women with no AMH measurement were excluded from this study. In analysis of pregnancy outcome, only the first embryo transfer was included. All the oocyte retrieval cycles and subsequent frozen embryo transfer if performed during this study period were analyzed. The data on IVF treatment and pregnancy outcome were retrieved from the electronic medical database. Diminished ovarian reserve was defined as serum AMH < 1 ng/ml or serum FSH > 15 IU/l and AFC < 4 on day 2–5 of the menstrual cycle [16]. Diagnosis of polycystic ovary syndrome (PCOS) was based on the modified Rotterdam criteria [17].

### Ethics approval and consent to participate

This study was approved by the Medical Ethics Committee of Shenzhen Maternity and Child Healthcare Hospital (SFYLS2019048). Given the retrospective nature of this study, the informed consent waived by the Medical Ethics Committee of Shenzhen Maternity and Child Healthcare Hospital.

### Blood samples and hormone test

Venous blood samples (about 3 ml) were drawn between day 2 and 5 of the menstrual period before ovarian stimulation. After collection, blood samples could clot at room temperature for 20 to 30 min. Fresh Serum was then separated by centrifugation (10 min at 1500 g) and analyzed within 8 h after blood collection. Serum FSH concentrations were measured using a standard chemiluminescence immunoassay (Beckman DXI800, Brea, California, USA) following the manufacturer ‘s instructions. The lower detection concentration was 0.1 mIU/ml. Serum AMH concentrations were measured by a one-step sandwich assay based on the acridinium direct chemiluminescence technology for use on iFlash 3000 immunoanalyzers (YHLO Biotech, Shenzhen, China). The assay limit of detection was 0.03 ng/ml for AMH provided by the manufacturer [18]. The intra- and interassay coefficients of variation were < 10% for all parameters.

### Controlled ovarian stimulation and embryo scoring

The controlled ovarian stimulation (COS) protocol for each patient was decided by the infertility physicians. The utility of gonadotropin-releasing hormone (GnRH) agonist or GnRH antagonist or no pituitary suppression regimen for COS procedures was based on a real-life approach. Follicular growth was monitored by serial ultrasound scans and serum hormone test. Triggering of ovulation was performed when at least one follicle reached a size of 17 mm or more. In extreme majority of cases, 10,000 IU human chorionic gonadotrophin was administered for triggering. Oocyte retrieval was scheduled 36 h later. The quality of a day 3 embryo was graded based on the number and symmetry of blastomeres and the amount of fragmentation. The scoring criteria of blastocyst quality included evaluation of the trophectoderm and the inner cell mass or inner cell mass (ICM), the degree of expansion of the blastocyst cavity and the status of the trophectoderm breakings out of the zona pellucida.

### Embryo transfer and pregnancy outcome follow-up

Embryo transfer was scheduled on cleavage-stage day 3 or 5 in all cases. Either natural, programmed or mild stimulation regimens was applied for endometrial preparation in frozen embryo cycles. The number of embryos transferred was based on the guidelines of the Chinese Society of Reproductive Medicine and American Society for Reproductive Medicine [19]. Dydrogesterone (10 mg tid) and P suppository (Cyclogest, 400 mg bid) or 8% Crinone gel (90 mg qd) were used for luteal support. Serum β human chorionic gonadotrophin (β-hCG) test was conducted 14 days after oocyte retrieval. Biochemical pregnancy was defined by a transient positive β-hCG test without the presence of gestational sac. Clinical pregnancy was diagnosed as the presence of gestational sac and heart beat detected by transvaginal ultrasonography 4 weeks after embryo transfer. Miscarriage was defined as the loss of clinical pregnancy prior to 24 weeks. Live birth was defined as the birth of at least one live infant after 24 weeks of gestation. The implantation rate, clinical pregnancy, miscarriage and ongoing pregnancy per embryo transfer were calculated.

### Data collection and statistics

A dedicated SPSS-based database was built for data retrieval and management. The data on patients’ demographics, COS, embryo transfer and pregnancy outcomes were recorded. A well-trained research nurse was responsible for the follow-up of pregnancy outcome and maintenance of the database. Chi-square test or Fisher’s exact test was applied to analyze the qualitative data. Independent-samples t test or Manne-Whitney U test was adapted to compare continuous variables. Shapiro-Wilk test was used to test the normality of continuous variables. The method to establish age-specific reference range for AMH was described in our previous report [20]. In details, the AMH concentrations at a given age had no Gaussian distribution with a mean and standard deviation varying greatly across ages. Therefore, the absolute AMH concentrations were log-transformed to be normal distribution. Subsequently, polynomial least-squares regression was applied to estimate the association between logAMH and age in the entire study cohort. The coefficients of determination (R^2^) in the linear regression model (*R*^2^ = 0.18) were greater than that in the quadratic regression model (*R*^2^ = 0.10). The linear regression equation was thus chosen to build the reference range of logAMH across ages. The 10^th^ and 90^th^ centile of the reference age were also calculated. An age-specific low AMH concentration was defined as an AMH level equal or lower than the 10^th^ centile at each age. Female age after 35 was directly related to increased risk of embryonic aneuploidy and decreased rare of cumulative live birth rates [21]. This study used the age 35 as the cutoff value to clarify the confounding of female age in the association of low AMH with pregnancy outcome.

## Results

**Fig. 1:**
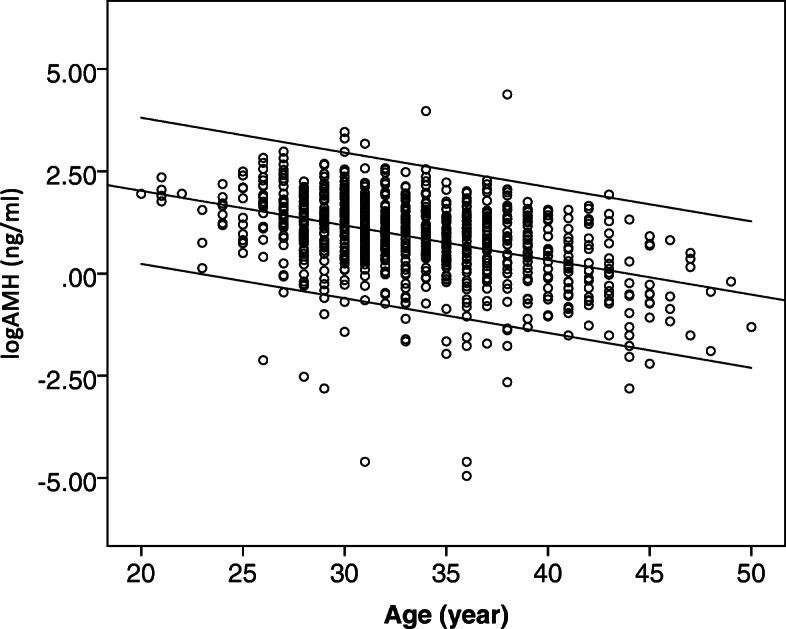
Scatter plot of AMH across ages and the estimated reference range with curves of 10^th^ and 90^th^ centiles

A total of 909 patients with AMH concentration available were included during the study period. In the total cohort, serum AMH concentrations were inversely correlated with age (Spearman r = − 0.43; *P* < 0.001). The Ln-transformation of serum AMH levels were used to build the age-related reference range, yielding the fitted curves for the mean LnAMH and the 10^th^ centile (Fig. 1 and Table 1). Their equations were as follows: mean LnAMH = (− 0.085 × age) + 3.714 and 10^th^ centile = (− 0.107 × age) + 3.39, respectively. Accordingly, 62 patients were categorized into the group of low AMH level. The comparison of clinical characteristics between patients with and without low AMH level were summarized in Table 2. The indications for IVF treatment were quite different between two groups. Diminished ovarian reserve was the main cause (88.7%) for IVF treatment in patients with low AMH levels.

**Table 1:** The estimated cut-off value of various centiles across ages

**Age, year**	**5**^**th**^ **centile**	**10**^**th**^ **centile**	**50**^**th**^ **centile**	**90**^**th**^ **centile**	**95**^**th**^ **centile**
20	2.81975	3.50425	7.54972	16.2655	20.2139
21	2.51792	3.14896	6.93721	15.2828	19.113
22	2.24839	2.82969	6.3744	14.3595	18.072
23	2.00772	2.5428	5.85725	13.492	17.0877
24	1.79281	2.28499	5.38205	12.6769	16.1571
25	1.6009	2.05332	4.94541	11.911	15.2771
26	1.42953	1.84514	4.54419	11.1914	14.445
27	1.27651	1.65806	4.17552	10.5153	13.6583
28	1.13987	1.48995	3.83677	9.88	12.9144
29	1.01786	1.33889	3.52549	9.2831	12.211
30	0.9089	1.20314	3.23947	8.7223	11.546
31	0.81161	1.08116	2.97665	8.1954	10.9171
32	0.72473	0.97154	2.73516	7.7002	10.3225
33	0.64716	0.87304	2.51326	7.235	9.7603
34	0.57788	0.78452	2.30936	6.7979	9.2287
35	0.51603	0.70498	2.122	6.3872	8.7261
36	0.46079	0.63351	1.94985	6.0014	8.2509
37	0.41146	0.56928	1.79166	5.6388	7.8015
38	0.36742	0.51156	1.6463	5.2981	7.3766
39	0.32809	0.45969	1.51274	4.9781	6.9748
40	0.29297	0.41308	1.39001	4.6773	6.5949
41	0.26161	0.3712	1.27724	4.3947	6.2358
42	0.23361	0.33357	1.17362	4.1292	5.8961
43	0.2086	0.29975	1.0784	3.8798	5.575
44	0.18627	0.26936	0.99091	3.6454	5.2714
45	0.16633	0.24205	0.91052	3.4251	4.9843
46	0.14853	0.21751	0.83665	3.2182	4.7128
47	0.13263	0.19545	0.76877	3.0238	4.4561
48	0.11843	0.17564	0.7064	2.8411	4.2134
49	0.10575	0.15783	0.64909	2.6695	3.9839
50	0.09443	0.14183	0.59643	2.5082	3.767

Data were the age-specific 5^th^, 10^th^, 50^th^, 90^th^ and 95^th^ centile of AMH values in unit of ng/ml

**Table 2:** Clinical baseline

Demographics	Patients with AMH > 10th centile (*n* = 847)	Patients with AMH ≤ 10th centile (*n* = 62)	
Female age, yrs	33.3 ± 5.0	33.8 ± 5.0	0.46
BMI, kg/m2	21.6 ± 2.9	21.6 ± 2.8	0.99
Duration of infertility, months	39 ± 32	40 ± 31	0.82
Menstrual period, days	30 ± 4	28 ± 3	0.003
Women with prior live birth, n (%)	202 (23.8%)	14 (22.6%)	0.46
Regular menstruation	728 (86%)	56 (90.3)	0.34
Ovary surgery history, n (%)	68 (8%)	4 (6.5%)	
Causes for infertility, n (%)			< 0.01
Tubal factor	359 (42.4%)	4 (6.5%)	
DOR	80 (9.4%)	55 (88.7%)	
PCOS	106 (12.5%)	0	
Endometriosis	61 (7.2%)	1 (1.6%)	
Adenomyosis	12 (1.4%)	0	
Male factor	173 (20.4%)	1 (1.6%)	
Combined	19 (2.2%)	1 (1.6%)	
Unexplained	37 (4.4%)	0	
AMH, ng/ml	3.8 ± 3.2	0.4 ± 0.3	< 0.01
FSH, IU/L	7.8 ± 3.4	14.7 ± 7.9	< 0.01
AFC	12 ± 7	5 ± 2	< 0.01

*BMI* Body mass index, *DOR* Decreased ovarian reserve, *PCOS* Polycystic ovary syndrome, *AMH* Anti-Mullerian hormone, *FSH* Follicle stimulation hormone, *AFC* Antral follicle count

The data on COS procedures and embryo culture were recapitulated in Table 3. A total of 1281 COS procedures were performed. In these 909 women, 680 women underwent 1 cycle of oocyte retrieval, 152 did 2 cycles of oocyte retrieval, 39 did 3 cycles of oocyte retrieval, 22 did 4 cycles of oocyte retrieval, 8 did 5 cycles of oocyte retrieval, 5 did 6 cycles of oocyte retrieval, 2 did 7 cycles of oocyte retrieval and 1 did 8 cycles of oocyte retrieval. All the analyzed parameters were significant different between patients with or without low AMH level (*p* < 0.01). The mean number of embryos available per cycle was significantly fewer in patients with low AMH level (0.8 ± 1.1) compared to patients without low AMH level (3.4 ± 2.7), *p* < 0.01. When further stratified according to age, the outcomes of ovarian stimulation and embryo culture in patients with or without low AMH level remained to be significantly different (Table 4).

**Table 3:** Outcome of ovary stimulation and embryo culture

	Retrieval cycles with AMH > 10th centile (*n* = 1134)	Retrieval cycles with AMH ≤ 10th centile (*n* = 147)	*P* value
Days of ovarian stimulation per cycle	10.1 ± 3.5	6.6 ± 5.2	< 0.01
Total gonadotropin dose (IU) per cycle	2690 ± 1368	1802 ± 1583	< 0.01
Estradiol level on trigger day (pg/ml)	2672 ± 1579	874 ± 827	< 0.01
Endometrial thickness on trigger day (mm)	9.4 ± 7.0	7.5 ± 2.8	< 0.01
Total number of oocytes per cycle	9.6 ± 6.9	2.2 ± 3.4	< 0.01
Number of MII oocytes per cycle	8.5 ± 6.4	1.9 ± 2.9	< 0.01
Total number of embryos available for transfer per cycle	3.4 ± 2.7	0.8 ± 1.1	< 0.01
Number of D5 or D6 blastocysts available for transfer per cycle	1.1 ± 1.7	0.1 ± 0.3	< 0.01

**Table 4:** Outcome of ovary stimulation and embryo culture by AMH percentile according to age

	**Age of patients < 35**		***P*** **value**
	**Retrieval cycles with AMH > 10th centile (*n*** **= 614)**	**Retrieval cycles with AMH **≤** 10th centile (*n*** **= 84)**	
Days of ovarian stimulation	11 ± 2.5	7.2 ± 5.1	< 0.01
Total gonadotropin dose (IU)	2850 ± 1302	1853 ± 1525	< 0.01
Estradiol level on trigger day (pg/ml)	3187 ± 1454	980 ± 882	< 0.01
Endometrial thickness on trigger day (mm)	10.2 ± 2.7	7.6 ± 2.9	< 0.01
Total number of oocytes retrieved	12.3 ± 6.5	2.7 ± 4.0	< 0.01
Number of MII oocytes retrieved	10.9 ± 6.2	2.4 ± 3.3	< 0.01
Total number of embryo available for transfer	4.3 ± 2.8	1.0 ± 1.2	< 0.01
Number of D5 or D6 blastocyst available for transfer	1.5 ± 1.9	0.1 ± 0.4	< 0.01
	**Age of patients ≥35**		
	**Retrieval cycles with AMH > 10th centile (*n*** **= 520)**	**Retrieval cycles with AMH ≤ 10th centile (*n*** **= 63)**	
Days of ovarian stimulation	9.1 ± 4.2	5.5 ± 5.2	< 0.01
Total gonadotropin dose (IU)	2500 ± 1420	1728 ± 1677	< 0.01
Estradiol level on trigger day (pg/ml)	2061 ± 1503	740 ± 735	< 0.01
Endometrial thickness on trigger day (mm)	8.5 ± 3.1	7.3 ± 2.7	< 0.01
Total number of oocytes retrieved	6.4 ± 5.9	1.5 ± 2.1	< 0.01
Number of mature oocytes retrieved	5.7 ± 5.4	1.3 ± 2.0	< 0.01
Total number of embryo available for transfer	2.4 ± 2.2	0.6 ± 0.8	< 0.01
Number of D5 or D6 blastocyst available for transfer	0.6 ± 1.2	0.1 ± 0.3	< 0.01

Table 5 presents the embryo transfer by AMH percentile according to patient age. A total of 1008 fresh or frozen embryo transfer cycles were performed. In patients younger than 35 years, transfer of D3 embryos was more frequently in patients with low AMH level compared to patients with no low AMH level (96.9 and 78.7%, respectively, *p* < 0.01). However, this difference was not detected in patients with age ≥ 35 years.

**Table 5:** Comparison of embryo transfer by AMH percentile according to patient age

	**Age of patients < 35**		***P*** **value**
	**Embryo transfer with AMH > 10th centile (*n*** **= 582)**	**Embryo transfer with AMH ≤ 10th centile (*n*** **= 32)**	
Fresh embryo transfer	191 (32.8%)	10 (31.2%)	0.85
Mean number of embryos transferred	1.9 ± 0.4	1.8 ± 0.4	0.17
One embryo transferred	94 (16.2%)	8 (25%)	0.19
Two embryos transferred	488 (83.9%)	24 (75%)	_
D3 embryo transfer	458 (78.7%)	31 (96.9%)	0.003
Blastocyst transfer	124 (21.3%)	1 (3.1%)	0.11
D5 blastocyst	93 (16%)	1 (3.1%)	_
D6 blastocyst	31 (5.3%)	0	_
	**Age of patients ≥35**		
	**Embryo transfer with AMH > 10th centile (*n*** **= 384)**	**Embryo transfer with AMH ≤ 10th centile (*n*** **= 10)**	
Fresh embryo transfer	123 (32.2%)	3 (30%)	1.00
Mean number of embryos transferred	1.8 ± 0.4	1.5 ± 0.5	0.02
One embryo transferred	74 (19.7%)	5 (50%)	0.019
Two embryos transferred	320 (81.3%)	5 (50%)	
D3 embryo transfer	304 (81.8%)	9 (90%)	0.47
Blastocyst transfer	72 (18.2%)	1 (10%)	_
D5 blastocyst	43 (11.4%)	0	__
D6 blastocyst	29 (7.7%)	1 (10%)	

Univariate regression analysis displayed a significant association of ongoing pregnancy with AMH concentration, oocyte number and female age (OR 1.11, 95% CI 1.06–1.16, OR = 1.05, 95%CI: 1.00–1.09 and OR = 0.89, 95%CI: 0.84–0.94, respectively). Multivariate logistic regression analysis showed that female age was independently associated with the rate of ongoing pregnancy (OR = 0.90, 95%CI: 0.855–0.953, *P* < 0.01) whereas no statistical significance was reached in the association of ongoing pregnancy with AMH concentration and oocyte number. We further evaluated the pregnancy outcomes following the entire cohort of 1008 fresh or frozen embryo transfer cycles according AMH level and age (Table 6). The mean female age in these 4 subgroups divided by age and AMH level in Table 6 were respectively 30.3 ± 2.6, 28.8 ± 2.7, 38.7 ± 3.0 and 38.5 ± 3.9 years. In patients younger than 35 years, significant differences between AMH subgroups were detected in clinical pregnancy per transfer (*p* = 0.003), miscarriage per clinical pregnancy (*p* = 0.015) and ongoing pregnancy per transfer (*p* = 0.001). Again, these differences between AMH subgroups were not reached in patients older than 35 years (Table 6).

**Table 6:** Pregnancy outcome after embryo transfer by AMH percentile according to patient age

	**Age of patients < 35**		***P*** **value**
	**Embryo transfer with AMH > 10th centile (*n*** **= 582)**	**Embryo transfer with AMH **≤** 10th centile (*n*** **= 32)**	
Positive hCG per transfer	62.5% (364/582)	37.5% (12/32)	0.005
Positive hCG per D3 embryo transfer	62.7% (287/458)	35.5% (11/31)	0.002
Positive hCG per blastocyst transfer	62.1% (77/124)	100% (1/1)	1.00
Biochemical pregnancy per transfer	4.6% (27/582)	6.3% (2/32)	0.62
Ectopic pregnancy per transfer	0.9% (5/582)	0	1.0
Clinical pregnancy per transfer	57% (332/582)	31.3% (10/32)	0.003
Miscarriage per clinical pregnancy	9% (30/332)	30% (3/10)	0.015
Ongoing pregnancy per transfer	51.9% (302/582)	21.9% (7/32)	0.001
Ongoing pregnancy per D3 embryo transfer	51.3% (235/458)	19.4% (6/31)	0.001
Ongoing pregnancy per blastocyst transfer	54% (67/124)	100% (1/1)	1.0
	**Age of patients** ≥**35**		
	**Embryo transfer with AMH > 10th centile (*n*** **= 384)**	**Embryo transfer with AMH **≤** 10th centile (*n*** **= 10)**	
Positive hCG per transfer	46.1% (177/384)	10% (1/10)	0.026
Positive hCG per D3 embryo transfer	43.6% (136/312)	11.1% (1/9)	0.005
Positive hCG per blastocyst transfer	56.9% (41/72)	0 (0/1)	0.43
Biochemical pregnancy per transfer	4.4% (17/384)	0	_
Ectopic pregnancy per transfer	0	0	_
Clinical pregnancy per transfer	41.7% (160/384)	10% (1/10)	0.049
Miscarriage per clinical pregnancy	18.1% (29/160)	0	1.00
Ongoing pregnancy per transfer	34.1% (131/384)	10% (1/10)	0.175
Ongoing pregnancy per D3 embryo transfer	31.1% (97/312)	11.1% (1/9)	0.285
Ongoing pregnancy per blastocyst transfer	47.2% (34/72)	0 (0/1)	1.00

Given that an AMH cut-off value of 1.0 ng/ml was used to define ovarian reserve in previous studies [16], Table 7 was added to correlate the fertility outcome with low AMH value (lower than 1.0 ng/ml). Statistical significance in clinical pregnancy per transfer and ongoing pregnancy per transfer (*p* = 0.001) was found between subgroup divided by absolute value of AMH according to patient age. In the subgroup of age after 35, women with AMH lower than 10^th^ centile had a lower clinical pregnancy per transfer and ongoing pregnancy per transfer compared to women with AMH lower than 1 ng/ml (10% vs 22.1, 10% vs 16.2%, respectively), though no statistical significance was found.

**Table 7:** Pregnancy outcome after embryo transfer by absolute value of AMH according to patient age

	**Age of patients < 35**		***P*** **value**
	**Embryo transfer with AMH ≥ 1.0 ng/ml (*n*** **= 576**)	**Embryo transfer with AMH < 1.0 ng/ml (*n*** **= 38**)	
Positive hCG per transfer	62.5% (360/576)	42.1% (16/38)	0.012
Positive hCG per D3 embryo transfer	62.5% (283/453)	35.5% (15/36)	0.014
Positive hCG per blastocyst transfer	62.6% (77/123)	50% (1/2)	1.00
Biochemical pregnancy per transfer	4.9% (28/576)	2.6% (1/38)	1.00
Ectopic pregnancy per transfer	0.9% (5/576)	0	1.00
Clinical pregnancy per transfer	56.8% (327/576)	39.5% (15/38)	0.038
Miscarriage per clinical pregnancy	9.2% (30/327)	20% (3/15)	0.167
Ongoing pregnancy per transfer	51.6% (297/576)	31.6% (12/38)	0.017
Ongoing pregnancy per D3 embryo transfer	50.8% (230/453)	30.6% (11/36)	0.02
Ongoing pregnancy per blastocyst transfer	54.5% (67/123)	50% (1/2)	1.00
	**Age of patients≥35**		
	**Embryo transfer with AMH ≥ 1.0 ng/ml (*n*** **= 326)**	**Embryo transfer with AMH < 1.0 ng/ml (*n*** **= 68)**	
Positive hCG per transfer	49.1% (160/326)	26.5% (18/68)	0.001
Positive hCG per D3 embryo transfer	46.5% (119/256)	27.7% (18/65)	0.006
Positive hCG per blastocyst transfer	58.6% (41/70)	0 (0/3)	0.08
Biochemical pregnancy per transfer	4.3% (14/326)	4.4% (3/68)	1.00
Clinical pregnancy per transfer	44.8% (146/326)	22.1% (15/68)	0.001
Miscarriage per clinical pregnancy	17.1% (25/146)	26.7% (4/15)	0.573
Ongoing pregnancy per transfer	34.1% (121/326)	16.2% (11/68)	0.001
Ongoing pregnancy per D3 embryo transfer	34.0% (87/256)	16.9% (11/65)	0.008
Ongoing pregnancy per blastocyst transfer	48.6% (34/70)	0 (0/3)	0.243

## Discussion

This study established an age-specific definition of low AMH concentration and further evaluated its impact on outcomes of assisted conceptions. The age-related 10^th^ centile of AMH concentrations was used as the cut-off value for low AMH level. The equation was as follows: 10^th^ centile = (− 0.107 × age) + 3.39. Based on this cut-off value, this study found that the pregnancy rate was significantly lower in women with low AMH concentration, especially when evaluated according to age.

AMH as a marker of ovarian reserve is well recognized and widely tested in clinical practice [2]. In accordance with previous studies, the present study showed the measurement of AMH was steadily decreased with advancing age [13, 14]. In addition, the AMH concentrations were well correlated with the number of oocytes retrieved during COS procedures [3]. In a study by Hamdine and colleagues, the authors further found that AMH had a higher accuracy for predicting ovarian response than female age and BMI [22].

However, the clinical utility of AMH for the prediction of pregnancy outcome remains controversial. In a previous study, Zhang et al. reported that the cumulative live birth in women with young age (< 35 year) and low AMH level (defined as 0-25th percentage) and those with advanced age (≥35 year) and low AMH level was respective 56.35%(1025/1819) and 20.11%(108/537) after the transfer of all embryos obtained in the first oocyte retrieval. In women with young age (< 35 year) and low AMH level, a plateau of cumulative live birth was reached after three embryos transfer attempts [23]. The present study found that the ongoing pregnancy rate was about 20% in women with young age (< 35 year) and low AMH level (defined as the 10th centile). Therefore, our study also provided evidence for young women with low AMH level to undergo more attempts of IVF treatments. This study presents that low AMH levels are negatively associated with implantation rate, clinical pregnancy and ongoing pregnancy rate in women younger than 35 years. Several studies also reported that AMH concentrations were associated with the implantation, clinical pregnancy and live birth after IVF treatment [5, 6]. Two studies respectively analyzed 603 and 892 patients and found that AMH levels were strongly associated with live birth after IVF conceptions [7, 24]. Other investigators, however, reported no or weak association of AMH with live birth [8–12]. For example, two recent systematic reviews and a large retrospective analysis of 85,062 cycles depicted AMH as a poor independent predictor for pregnancy outcome in IVF conceptions. Another study also showed that AMH provided little additional value for predicting 1-year cumulative live birth rate in GnRH antagonist treatment cycles [25]. The discrepancy among these studies may be mainly due to the confounding impact of age. Given that AMH levels decreases steadily with age, the cut-off value for low AMH may thus be evaluated according to age. Nevertheless, most studies adopted a uniform cut-off value of low AMH level for all women. In addition, the option of cut-off value of low AMH level are slightly different in these studies ranging from 0.4 to 2.7 ng/ml [12].

The main strength of this study is the application of age-specific definition for low AMH level. The age-related cut-off value may be not as clinically pragmatic as the single threshold for all ages. However, it is helpful to minimize the mixed effect of age on the association between low AMH level and pregnancy outcome following IVR treatment. In addition, the third-generation automated AMH assay was employed for the measures in the present study [26]. Several studies show that the automated AMH assay is more sensitive than the Gen-II ELISA kit [27]. The automated AMH assay avoids getting lost in the AMH values, especially the extremely low values and enables establishing more accurate reference [28–30].

There are several limitations in this study. First, the measurement of AMH was introduced into our center from 2016. Therefore, the circulating AMH levels was not tested in all the women undergoing IVF treatment at the beginning. This may result in a bias of patient selection. Second, the small sample size of women with low AMH level and older than 35 years limits a firm conclusion on the association between low AMH level and pregnancy outcome in this population. A debate on offering IVF treatment for these patients continues. In addition, the optimal method to determine the clinically most useful low AMH is to use AMH value from each specific age as continuous variable and assess its correlation with outcome parameters. However, this methodology would require a dataset too large to be practical for a single center study. Therefore, we adopted a more pragmatic methodology in this study. In brief, the age-specific reference of AMH was built by polynomial least-squares regression analysis. Given that around 10–30% of patients presenting to doctors with infertility were diagnosed as decreased ovarian reserve based on an AMH level around 1.0 ng/ml or 10th centile [31–33], this study also chose the age-specific 10^th^ centile as the cut-off value of low AMH. However, the sample size of patients with extremely low AMH level (5^th^ centile) were quite small in the present study, which not only prevents meaningful analysis, but also obscure the significant association between low AMH and pregnancy outcome. Large studies with more patients with low AMH level are required to confirm our findings. The dichotomization of the outcomes (age and AMH), though clinically useful, may lead to a considerable loss of statistical power of the analysis, missing information and potentially biased effect estimates on individual patient. In particular, dichotomization leads to a considerable loss of power and incomplete correction for confounding factors. Moreover, Extended blastocyst-stage embryo culture in women with advanced age is full of debate. Several studies demonstrated that blastocyst development rate and implantation rate after blastocyst transfer was negatively related to increasing female aging [33, 34]. In a Cochrane systematic review, the evidence of blastocyst culture in women with advanced age is quite limited [35, 36]. In our practice, women with advanced age are often afraid of the failure in blastocyst culture and losing the opportunity to transfer cleavage-stage embryos. As a result, the blastocyst culture is less performed in women with advanced age than young women. Noticeably, it is well recognized that serum AMH is an excellent indicator of the number of oocytes and embryos per ovarian stimulation cycle. The higher serum AMH is, the more oocytes and embryos yields per ovarian stimulation cycle. In this sense, AMH may foresee cumulative pregnancy outcome during IVF treatment. Large and perspective studies are required to show the association between centiles-different AMH and cumulative live birth in patients at same ages.

## Conclusion

Our findings suggest that low AMH concentrations are associated with poor ovarian stimulation and pregnancy rate in women younger than 35 years. Large studies are required to investigate the predictive value of age-specific low AMH levels for pregnancy outcomes in women older than 35 years.

## Data Availability

The raw dataset analyzed in the current study are available from the corresponding author on reasonable request.

## Abbreviations

AFC: Antral follicle count
AMH: Anti-Mullerian hormone
β-hCG: β human chorionic gonadotrophin
BMI: Body mass index
COS: Controlled ovarian stimulation
DOR: Decreased ovarian reserve
D3: Day 3
D5: Day 5
D6: Day 6
FSH: Follicle stimulation hormone
IU: International unit
IVF: In vitro fertilization
PCOS: Polycystic ovary syndrome
TGF-β: Transforming growth factor-β
